# Parental occupational exposures in wood-related jobs and risk of testicular germ cell tumours in offspring in NORD-TEST a registry-based case–control study in Finland, Norway, and Sweden

**DOI:** 10.1007/s00420-021-01818-4

**Published:** 2021-12-01

**Authors:** Sara Corbin, Kayo Togawa, Joachim Schüz, Charlotte Le Cornet, Beatrice Fervers, Maria Feychting, Pernilla Wiebert, Johnni Hansen, Susanne Oksbjerg Dalton, Kristina Kjærheim, Karl-Christian Nordby, Ragnhild Strand Østrem, Niels E. Skakkebæk, Sanni Uuksulainen, Eero Pukkala, Ann Olsson

**Affiliations:** 1 Environment and Lifestyle Epidemiology Branch, International Agency for Research on Cancer (IARC/WHO), 150 cours Albert Thomas, 69372 Lyon Cedex 08, France; 2 Deutsches Krebsforschungszentrum (DKFZ), Heidelberg, Germany; 3 Prevention Cancer Environment Department, Centre Léon Bérard, University of Lyon, Lyon, France; 4 Inserm UMR 1296 Radiations: Defence, Health, Environment, Lyon, France; 5 Institute of Environmental Medicine, Karolinska Institutet, Stockholm, Sweden; 6 Danish Cancer Society Research Center, Copenhagen, Denmark; 7 Department of Research, Cancer Registry of Norway, Oslo, Norway; 8 National Institute of Occupational Health, Oslo, Norway; 9 Department of Growth and Reproduction and International Center for Research and Training in Endocrine Disruption of Male Reproduction and Child Health (EDMaRC), University of Copenhagen, Copenhagen, Denmark; 10 Finnish Institute of Occupational Health (FIOH), Helsinki, Finland; 11 Finnish Cancer Registry, Institute for Statistical and Epidemiological Cancer Research, Helsinki, Finland; 12 Finland School of Health Sciences, University of Tampere, Tampere, Finland

**Keywords:** Testicular neoplasms, Testicular germ cell tumours, Nordic countries, Occupational exposure, Wood-related jobs, Prenatal exposure delayed effects

## Abstract

**Objective:**

We assessed the association between parental prenatal exposures in wood-related jobs and risk of testicular germ cell tumours (TGCT) in offspring.

**Methods:**

NORD-TEST, a registry-based case–control study in Sweden, Finland and Norway, included 8112 TGCT cases diagnosed at ages 14–49 years between 1978 and 2012 with no history of prior cancer, and up to four controls matched to each case on year and country of birth. Parents of cases and controls were identified via linkages with the population registries and their occupational information was retrieved from censuses. The Nordic Occupational Cancer Study Job-Exposure Matrix was used to assign occupational exposures to each parent. Logistic regression models were used to estimate odds ratios (OR) and 95% confidence intervals (CI).

**Results:**

Maternal wood-related job was not associated with the risk of TGCT in offspring (OR 1.08, CI 0.55–2.14), while paternal wood-related job was associated with a decreased risk of TGCT in offspring (OR 0.85, CI 0.75–0.96). None of the specific wood-related jobs, such as upholsterers, sawyers, or construction carpenters, were significantly associated with a risk of TGCT. Only exception was observed in a sensitivity analysis which showed an increased risk in the small group of sons of fathers working as ‘cabinetmakers and joiners’ the year before conception (OR of 2.06, CI 1.00–4.25).

**Conclusion:**

This large-scale NORD-TEST analysis provided no evidence of an association between parental prenatal exposures in wood-related jobs and TGCT in sons.

**Supplementary Information:**

The online version contains supplementary material available at 10.1007/s00420-021-01818-4.

## Introduction

Testicular cancer represents less than 1% of cancers in men worldwide, yet it is the most frequent malignancy in men between the age of 15 and 44 years in high-income countries (Ferlay et al. 2021). The incidence has been rising rapidly over the last decades in various regions of the world, and more markedly in Southern Europe and Latin America (Znaor et al. 2014). The highest incidence rates are still found in the Northern European countries (Ferlay et al. 2021). Testicular germ cell tumours (TGCT) associated with germ-cell neoplasia in situ (GCNIS) are by far the most frequent type of testicular cancer (Gilligan et al. 2019). There are two main types of TGCT: seminoma and non-seminoma, where the non-seminoma tumours are identified according to what type of cells they are made of. With effective treatment, the overall five-year survival rate is 97% (Baird et al. 2018), although the treatment can cause impairment of spermatogenesis and also lead to secondary cancers (Parekh et al. 2020; Schaffar et al. 2019).

The links between testicular cancer and early life disorders of the male genitalia (e.g., cryptorchidism, hypospadias, impaired spermatogenesis) that are associated with fetal androgen insufficiency (Pleskacova et al. 2010) have led to the hypothesis of a testicular dysgenesis syndrome (TDS) of fetal origin (Moch et al. 2016; Skakkebaek et al. 2001). In line with the TDS hypothesis, there are numerous studies showing that the GCNIS cells (the precursor cells of testicular cancer) express embryonic markers similarly to normal fetal germ cells, and they represent transformed fetal cells (gonocytes) (Skakkebaek et al. 1987; Sonne et al. 2009).

The geographic and rapid time variations in testicular cancer incidence make exposure to environmental factors one of the most plausible reasons for the worldwide increase in TGCT incidence (Schmiedel et al. 2010). Moreover, studies showed that immigrants kept the incidence rate of TGCT of their home country after relocation whereas their sons had the same incidence rate as people in the host country (Huyghe et al. 2007). This quick shift in incidence rate is in favour of environmental risk factors related to perinatal development of the testis.

Exogenous environmental exposures during adulthood have been identified as plausible risk factors, such as household adult exposure to pesticides (McGlynn and Trabert 2012), firefighting and aircraft maintenance (McGlynn and Trabert 2012), and marijuana smoking (Lacson et al. 2012), but with remaining uncertainty. Furthermore, prenatal environmental exposures remain a solid hypothesis because of the foetal phenotype of the precursor cells, GCNIS, and the histopathology of TGCT (Béranger et al. 2013). Maternal exposure to exogenous oestrogens during pregnancy may induce male reproductive tract abnormalities, and certain occupational exposures among fathers have been suggested to cause genetic alteration in sperm before conception which could affect cancer susceptibility in the child (Feychting et al. 2001; Sharpe and Skakkebaek 1993).

A previous analysis of the NORD-TEST Study in Finland, Norway and Sweden (Togawa et al. 2016) studied parental occupational exposure to heavy metals and welding fumes in relation to TGCT in sons. The overall results showed no association, except for paternal exposure to chromium with high probability/high intensity. Parental prenatal occupational exposure to solvents also showed no significant association overall, but a sensitivity analysis restricted to parents with census information from the year (± 1 year) of childbirth, to minimize exposure misclassification, showed an association with maternal exposure to aromatic hydrocarbon solvents (Le Cornet et al. 2017). In NORD-TEST Denmark, where the complete work history prior to birth was available, but was accessed based on an industry level rather than on job level as in the other Nordic countries, no notable associations were found for either parental exposure to metals or solvents except for paternal exposure to “heavy metal other than lead” which was significantly associated with an increased risk of TGCT in sons (Olsson et al. 2018). Most of the fathers exposed to “heavy metals other than lead” (*N* = 144) were exposed to both chromium and toluene, and held a wood-related job, such as “wooden and upholstered furniture factories”, “manufacture of building articles”, or “sawmills (Olsson et al. 2018). Wood dust exposure is associated with different exposure scenarios and compounds depending on the industry sector, tasks and wood preservatives (e.g. formaldehyde, chromium, organochlorine compounds) (Ghelli et al. 2021; Vallières et al. 2015). Exposure to wood dust as well and formaldehyde have been shown to induce oxidative stress in occupationally exposed subjects even at levels below current occupational exposure limits (Ghelli et al. 2021). In experimental studies, several of these compounds have shown to induce oxidative stress in testicular tissue and germ cells (Duong et al. 2011; Pereira et al. 2021; Sujatha et al. 2001). Moreover, experimental studies conducted in adult as well as transgenerational animals, suggest testicular oxidative stress to be involved in testicular dysfunction, germ cell damage and altered organ responses to endocrine stimuli, as well as genotoxicity (Archibong et al. 2018; Dutta et al. 2021).

The present analysis aimed to (1) investigate the associations between parental occupation in wood-related jobs (and specific exposures within these jobs) before and closest to the year of childbirth and the risk of TGCT in sons, and (2) investigate the associations found in Denmark for chromium and toluene among wood-workers from Sweden, Finland, and Norway.

## Materials and methods

The NORD-TEST Study is a registry-based case–control study conducted in Sweden, Norway, Finland and Denmark. However, in this analysis, Denmark was excluded because we were unable to harmonize the occupational data due to a different source and format. The relevant national data protection/ethics committees, as well as the International Agency for Research on Cancer’s (IARC) ethics committee provided approval for NORD-TEST (project no.12-10).

### Study population

Cases are men diagnosed with TGCT at ages 14–49 years between 1978 and 2012 (end of the NORD-TEST data collection) without history of previous neoplasm (except non-melanoma skin cancer). Cases had to be born in Sweden, Finland and Norway, and at least one of the parents had to be economically active to be included in the study. Cases were identified from the population-based cancer registries established in the 1950s and 1960s in the Nordic countries (Pukkala et al. 2018), while control subjects were randomly chosen from the respective central population registry and were matched to cases on year and country of birth. Missing data on occupation for both parents occurred for 948 cases and 4574 controls. After these exclusions, the study sample included a total of 8112 cases and 26,264 controls for the analysis.

### Data collection

A unique personal identification number obtained from birth in the Nordic countries allowed linkage of the population-based registries (birth, hospital discharge, cancer, and demographic information registries) and census information in each country to retrieve the relevant information including personal and family history of cancer, cryptorchidism, hypospadias, and inguinal hernia, as well as parents’ age at childbirth and occupations for each case and control. Censuses including information on jobs held were conducted every 5 years in Sweden and Finland, and every 10 years in Norway. To estimate prenatal parental exposure, censuses closest and prior to the child’s birth were selected between 1960 and 1990 in Sweden, between 1960 and 1980 in Norway, and between 1970 and 1990 in Finland. We assumed that parents held the same job at conception/during pregnancy as when filling in the census closest and prior to the child’s birth.

### Exposure assessment

Wood-related jobs were defined as those jobs that are assigned wood dust in the Nordic Occupational Cancer Study Job-Exposure Matrix (NOCCA-JEM), namely upholsterers, timbermen, sawyers, plywood and fibreboard workers, construction carpenters, wooden boat builders, etc., bench carpenters, cabinetmakers and joiners, etc., woodworking machine operators, etc., wooden surface finishers, and woodworking occupations not elsewhere classified (n.e.c.). The NOCCA-JEM assigns exposures to these jobs via two quantitative variables: the estimated proportion of people exposed in a specific job (P) and the mean level of exposure (L) both of which were estimated for four periods (1945–1959, 1960–1975, 1975–1984, and 1985–1994) (Kauppinen et al. 2009). Parents were considered as exposed when the product of P and L was above 0, and non-exposed when it was equal to 0. Specific exposures studied included formaldehyde, aliphatic and alicyclic hydrocarbon solvents, aromatic hydrocarbon solvents and specifically benzene and toluene, methylene chloride, 1,1,1-trichloroethane, chromium, quartz dust, asbestos, insecticides, and fungicides. Supplementary Table 1 lists wood-related jobs and other jobs where these exposures are found according to the NOCCA-JEM.

### Statistical analysis

First, we estimated the associations between specific parental wood-related jobs and the risk of TGCT in sons. For this, we used conditional logistic regression models to estimate odd ratios (ORs) and corresponding 95% confident intervals (CIs) in the matched case–control pairs. The wood-related jobs were entered in the model one by one using a binary exposure variable. None of the pre-defined risk factors and potential confounders (history of cryptorchidism, hypospadias, inguinal hernia, family history (brother or father) of TGCT, and parental age at childbirth) changed the OR for the wood-related jobs, and were therefore not included in the final models.

Second, we estimated the associations between specific exposures present in wood-related jobs and the TGCT risk in sons among those whose parents held wood-related jobs before and around childbirth. The latter analysis used unconditional logistic regression models to estimate ORs and CIs adjusted for the matching variables (year and country of birth). Specific wood-related exposures were also categorized as a binary exposure variable (exposed vs. non-exposed). To address our second objective (to investigate the associations found in Denmark for chromium and toluene), we generated a variable including three categories: parents exposed to both chromium and toluene (“Both”), parents exposed to either chromium or toluene (“Either”), and parents exposed to none of the agents (“Non-exp”) which was used as the reference category.

Additionally, for both analyses of wood-related jobs and specific exposures, we performed analyses stratified by subtype of TGCT, i.e. seminoma and non-seminoma.

Finally, a sensitivity analysis was conducted to minimize exposure misclassification, by restricting the study sample to parents with census information during the year (± 1 year) of childbirth, and parents with the same job in the census before and after birth.

All statistical tests were two-sided (alpha = 0.05) and performed using R version 3.5.0. ORs were not estimated if the number of exposed cases was less than 5 for all TGCT. This applied to fathers being timbermen (1 case), plywood & fireboard workers (1 case), wooden boat builders (4 cases), and wooden surface finishers (0 cases), and for mothers only, “any wood job” was analysed. For the specific chemical exposures, this applied to insecticide exposures in fathers (1 case) and most of the exposures among mothers.

## Results

The analysis included 8112 cases and 26,264 matched controls. The main characteristics of the study sample are described in Table 1. The proportion of non-seminoma cases was 55% with the median age of 26.7 years, whereas that of seminoma was 45% with the median age of 32.2 years. Mother’s mean age at childbirth was 27.2 years in both cases and controls, and father’s mean age at childbirth was 30.3 years in cases and 30.2 years in controls. The median time between the last census and childbirth was about 3 years for both cases and controls. Mother’s job was missing for 12,719 (48.4%) controls and 3935 (48.5%) cases, while father’s job was missing for 3670 (14.0%) controls and 1123 (13.8%) cases.

**Table 1:** Study population characteristics

Characteristics	Categories	Cases (*N* = 8112)	Controls (*N* = 26,264)
		*N* (%)	*N* (%)
Country	Sweden	3915 (48.3)	13,145 (50.0)
	Finland	1034 (12.7)	4030 (15.3)
	Norway	3163 (39.0)	9089 (34.6)
Birth year	1960–1964	1651 (20.4)	5352 (20.4)
	1965–1969	1489 (18.4)	4490 (17.1)
	1970–1974	1926 (23.7)	6398 (24.4)
	1975–1979	1518 (18.7)	4848 (18.5)
	1980–1984	974 (12.0)	3258 (12.4)
	1985–1989	433 (5.3)	1487 (5.7)
	> 1990	121 (1.5)	431 (1.6)
Age of father at childbirth, years	14–19	85 (1.1)	250 (1.0)
	20–29	4050 (49.9)	13,205 (50.3)
	30–39	3168 (39.1)	10,353 (39.4)
	40 +	732 (9.0)	2285 (8.7)
	Missing	77 (1.0)	171 (0.7)
Age of mother at childbirth, years	14–19	440 (5.4)	1500 (5.7)
	20–29	5155 (63.6)	16,625 (63.3)
	30–39	2314 (28.5)	7484 (28.5)
	40 +	192 (2.4)	647 (2.5)
	Missing	11 (0.1)	8 (0.0)
Inguinal hernia	No	6991 (86.2)	22,820 (86.9)
	Yes	108 (1.3)	414 (1.6)
	Missing	1013 (12.5)	3030 (11.5)
Hypospadias	No	7062 (87.1)	23,187 (88.3)
	Yes	37 (0.5)	47 (0.2)
	Missing	1013 (12.5)	3030 (11.5)
Cryptorchidism	No	7008 (86.4)	23,115 (88.0)
	Yes	91 (1.1)	119 (0.5)
	Missing	1013 (12.5)	3030 (11.5)
Father had TGCT	No	8043 (99.2)	26,192 (99.7)
	Yes	69 (0.8)	72 (0.3)
Brother had TGCT	No	8055 (99.3)	26,230 (99.9)
	Yes	57 (0.7)	34 (0.1)

As expected, hypospadias and cryptorchidism were associated with increased risk of TGCT. Family history of TGCT was also associated with increased risk of TGCT in sons, with a stronger association found with history of TGCT in brothers compared to that in fathers. Personal history of inguinal hernia was not associated with the risk of TGCT in our study (Le Cornet et al. 2017).

### Wood-related jobs

Very few mothers (0.2%) and 4.8% of fathers worked in wood-related jobs prior to birth. Table 2 shows TGCT risks in sons overall and by TGCT subtype in relation to parental wood-related jobs. Sons of fathers working in “any wood-related job” had a lower risk of TGCT (OR 0.85, CI 0.75–0.96), as well as sons of fathers working in “woodworking occupations, n.e.c.” (OR 0.62, CI 0.40–0.97). None of the specific father’s wood-related jobs showed a significant association with non-seminoma TGCT in sons, while father’s job in “any wood-related job” and as “construction carpenter” was associated with a decreased risk of seminomas in sons (OR 0.77, CI 0.63–0.93 and OR 0.74, CI 0.56–0.96, respectively).

**Table 2:** Risk of all types of testicular germ cell tumours (TGCT) and subtypes of TGCT in relation to parental wood-related occupations

		All TGCT			Seminoma			Non-seminoma		
		Controls *N* (%^a^)	Cases *N* (%^a^)	OR (95% CI)	Controls *N* (%^a^)	Cases *N* (%^a^)	OR (95% CI)	Controls *N* (%^a^)	Cases *N* (%^a^)	OR (95% CI)
*Fathers’ wood jobs*										
Any wood jobs	No	21,281 (81.0)	6641 (81.9)		9666 (81.2)	3037 (82.4)		11,615 (80.8)	3604 (81.4)	
	Yes	1313 (5.0)	348 (4.3)	0.85 (0.75–0.96)	604 (5.1)	144 (3.9)	0.77 (0.63–0.93)	709 (4.9)	204 (4.6)	0.92 (0.78–1.09)
Upholsterers	No	22,550 (85.9)	6976 (86.0)		10,252 (86.2)	3176 (86.1)		12,288 (85.6)	3800 (85.9)	
	Yes	44 (0.2)	13 (0.2)	0.99 (0.53–1.87)	18 (0.2)	5 (0.1)	0.88 (0.32–2.40)	26 (0.2)	8 (0.2)	1.08 (0.48–2.44)
Sawyers	No	22,518 (85.7)	6972 (86.0)		10,242 (86.1)	3175 (86.1)		12,276 (85.4)	3797 (85.8)	
	Yes	76 (0.3)	17 (0.2)	0.88 (0.51–1.51)	28 (0.2)	6 (0.2)	0.81 (0.33–2.02)	48 (0.3)	11 (0.2)	0.92 (0.47–1.81)
Construction carpenters	No	21,903 (83.4)	6797 (83.8)		9955 (83.7)	3107 (84.3)		11,948 (83.2)	3690 (83.4)	
	Yes	691 (2.6)	192 (2.4)	0.87 (0.74–1.03)	315 (2.6)	74 (2.0)	0.74 (0.56–0.96)	376 (2.6)	118 (2.7)	0.99 (0.79–1.23)
Bench carpenters	No	22,427 (85.4)	6944 (85.6)		10,198 (85.7)	3159 (85.7)		12,229 (85.1)	3785 (85.5)	
	Yes	167 (0.6)	45 (0.6)	0.87 (0.62–1.23)	72 (0.6)	22 (0.6)	1.07 (0.64–1.79)	95 (0.7)	23 (0.5)	0.74 (0.46–1.18)
Cabinetmakers and joiners	No	22,521 (85.8)	6962 (85.8)		10,229 (86.0)	3168 (85.9)		12,292 (85.6)	3794 (85.7)	
	Yes	73 (0.3)	27 (0.3)	1.16 (0.73–1.85)	41 (0.3)	13 (0.4)	1.01 (0.52–1.96)	32 (0.2)	14 (0.3)	1.35 (0.70–2.57)
Wood machine operators	No	22,501 (85.7)	6967 (85.9)		10,229 (86.0)	3170 (86.0)		12,272 (85.4)	3797 (85.8)	
	Yes	93 (0.3)	22 (0.3)	0.76 (0.47–1.24)	41 (0.3)	11 (0.3)	0.79 (0.39–1.61)	52 (0.4)	11 (0.2)	0.73 (0.37–1.43)
Woodworking occupations n.e.c	No	22,462 (85.5)	6964 (85.8)		10,199 (85.7)	3170 (86.0)		12,263 (85.4)	3794 (85.7)	
	Yes	132 (0.5)	25 (0.3)	0.62 (0.40–0.97)	71 (0.6)	11 (0.3)	0.56 (0.29–1.07)	61 (0.4)	14 (0.3)	0.69 (0.37–1.29)
*Mothers’ wood jobs*										
Any wood jobs	No	13,499 (51.4)	4163 (51.3)		5761 (48.4)	1772 (48.1)		7738 (53.9)	2391 (54.0)	
	Yes	46 (0.2)	14 (0.2)	1.08 (0.55–2.14)	19 (0.2)	2 (0.1)	0.52 (0.11–2.39)	27 (0.2)	12 (0.3)	1.39 (0.64–3.06)

*CI* confidence interval; *N* number; *OR* odds ratio; *n.e.c.* not elsewhere classified

^a^Mothers and fathers not working were excluded

We observed no association with risk of TGCT in sons of mothers working in wood-related jobs (OR 1.08, CI 0.55–2.14). The number of mothers working in wood-related jobs was too small to conduct an analysis of specific jobs.

### Specific exposures in wood-related jobs

None of the exposures occurring in the wood-related jobs, for either parent, was significantly associated with risk of TGCT in offspring (Table 3). There was also no association of TGCT with combined exposure to chromium and toluene in fathers (OR 0.98, CI 0.86–1.12) or mothers (OR 1.38. CI 0.69–2.78). An almost three-fold increased OR was seen with maternal exposure to aromatic hydrocarbon solvents but due to the small numbers the confidence interval was wide and including the null effect (OR 2.84, CI 0.97–12.05).

**Table 3:** Risk of testicular germ cell tumours (TGCT) in offspring in relation to specific chemical exposures in parents working in wood-related jobs

		Fathers working in wood-related jobs			Mothers working in wood-related jobs		
Exposures^b^		Controls *N* = 1313 *N* (%)	Cases *N* = 348 *N* (%)	OR (95% CI)^a^	Controls *N* = 46 *N* (%)	Cases *N* = 14 *N* (%)	OR (95% CI)^a^
Aliphatic and alicyclic hydrocarbon solvents	Non-exp	1268 (96.6)	335 (96.3)	1 (ref)	37 (80.4)	11 (78.6)	1 (ref)
	Exp	45 (3.4)	13 (3.7)	1.18 (0.62–2.21)	9 (19.6)	3 (21.4)	NA
Aromatic hydrocarbon solvents	Non-exp	1102 (83.9)	290 (83.3)	1 (ref)	32 (69.6)	7 (50.0)	1 (ref)
	Exp	211 (16.1)	58 (16.7)	1.29 (0.91–1.84)	14 (30.4)	7 (50.0)	2.84 (0.97–12.05)
Toluene	Non-exp	1211 (92.2)	322 (92.5)	1 (ref)	33 (71.7)	10 (71.4)	1 (ref)
	Exp	102 (7.8)	26 (7.5)	0.89 (0.56–1.39)	13 (28.3)	4 (28.6)	NA
Benzene	Non-exp	1287 (98.0)	340 (97.7)	1 (ref)	43 (93.5)	14 (100.0)	1 (ref)
	Exp	26 (2.00)	8 (2.3)	1.18 (0.53–2.64)	3 (6.5)	0 (0.0)	NA
1,1,1-trichloroethane	Non-exp	1297 (98.88)	343 (98.6)	1 (ref)	42 (91.3)	13 (92.9)	1 (ref)
	Exp	16 (1.2)	5 (1.4)	1.25 (0.45–3.45)	4 (8.7)	1 (7.1)	NA
Methylene chloride	Non-exp	1269 (96.6)	335 (96.3)	1 (ref)	38 (82.6)	11 (78.6)	1 (ref)
	Exp	44 (33.5)	13 (3.7)	1.20 (0.64–2.26)	8 (17.4)	3 (21.4)	NA
Chromium	Non-exp	1072 (81.6)	295 (84.8)	1 (ref)	23 (50.0)	11 (78.6)	1 (ref)
	Exp	241 (18.4)	53 (15.2)	0.74 (0.53–1.04)	23 (50.0)	3 (21.4)	NA
Exposure to chromium and toluene^c^	Non-exp	1028 (78.3)	282 (81.0)	1 (ref)	15 (32.6)	8 (57.1)	1 (ref)
	Either	227 (17.3)	53 (15.2)	0.85 (0.61–1.19)	26 (56.5)	5 (35.7)	0.33 (0.07–1.49)
	Both	58 (4.4)	13 (3.7)	0.67 (0.36–1.26)	5 (10.9)	1 (7.1)	NA
Formaldehyde	Non-exp	895 (68.2)	236 (67.8)	1 (ref)	23 (50.00)	7 (50.00)	1 (ref)
	Exp	418 (31.8)	112 (32.2)	0.98 (0.76–1.27)	23 (50.0)	7 (50.00)	0.92 (0.26–3.26)
Quartz dust	Non-exp	622 (47.4)	156 (44.8)	1 (ref)	45 (97.8)	12 (85.7)	1 (ref)
	Exp	691 (52.6)	192 (55.2)	1.07 (0.84–1.36)	1 (2.2)	2 (14.3)	NA
Asbestos	Non-exp	622 (47.4)	156 (44.8)	1 (ref)	45 (97.8)	12 (85.7)	1 (ref)
	Exp	691 (52.6)	192 (55.2)	1.07 (0.84–1.36)	1 (2.2)	2 (14.3)	NA
Insecticides	Non-exp	1300 (99.0)	347 (99.7)	1 (ref)	38 (82.6)	14 (100.0)	1 (ref)
	Exp	13 (1.0)	1 (0.3)	NA	8 (17.4)	0 (0.0)	NA
Fungicides	Non-exp	1224 (93.2)	330 (93.2)	1 (ref)	31 (67.4)	13 (92.9)	1 (ref)
	Exp	89 (6.8)	18 (6.8)	0.86 (0.50–1.47)	15 (32.6)	1 (7.1)	NA

*CI* confidence interval; *N* number; *OR* odds ratio; *n.e.c.* not elsewhere classified; *NA* not applicable

^a^ORs and CIs were estimated using non conditional regression adjusted on birth year and country

^b^Non-exp = Not exposed to the agent; Exp = exposed to the agent

^c^Non-exp = neither exposed to chromium nor toluene; Either = Exposed to either chromium or toluene; Both = Exposed to both chromium and toluene

### Sensitivity analysis

The sensitivity analysis restricted to the subjects with census information during the year (± 1 year) of childbirth, or the same job in the census before and after birth, showed an increased risk of TGCT for sons of fathers working as “cabinetmakers and joiners” (OR 2.06, CI 1.00–4.25), but was based on a small number of exposed fathers (*n* = 41) (Table 4). No other significant association between fathers working in wood-related jobs and sons developing TGCT was observed. The ORs for fathers working in “any wood-related job” and “woodworking occupations, not elsewhere classified (n.e.c.)” were similar (OR 0.87, CI 0.73–1.04 and OR 0.47, CI 0.20–1.11, respectively) to those from the main analyses (Table 2).

**Table 4:** Sensitivity analysis (restricting the study sample to cases and controls with information on parental occupations in the year of or the year before subject's birth) of risk of all types of testicular germ cell tumours (TGCT) and subtypes of TGCT in relation to parental wood-related occupation

		All TGCT^b^			Seminoma^b^			Non-seminoma^b^		
		Controls *N* (%^a^)	Cases *N* (%^a^)	OR (95% CI)	Controls *N* (%^a^)	Cases *N* (%^a^)	OR (95% CI)	Controls *N* (%^a^)	Cases *N* (%^a^)	OR (95% CI)
*Fathers exposed to wood*										
Any wood job	No	13,087 (91.0)	3950 (92.2)		4122 (90.4)	1759 (92.6)		5179 (89.5)	2191 (91.9)	
	Yes	776 (5.4)	208 (4.9)	0.87 (0.73–1.04)	241 (5.3)	88 (4.6)	0.80 (0.61–1.05)	293 (5.1)	413 (5.0)	0.93 (0.73–1.17)
Total		13,863 (96.4)	4158 (97.06)		4363 (95.7)	1847 (97.3)		5472 (94.6)	2311 (96.9)	
Upholsterers	No	13,832 (96.2)	4149 (96.8)							
	Yes	31 (0.2)	9 (0.2)	0.78 (0.33–1.85)						
Sawyers	No	13,822 (96.4)	4151 (96.9)							
	Yes	41 (0.3)	7 (0.2)	0.76 (0.33–1.74)						
Construction carpenters	No	13,379 (93.1)	4024 (93.9)							
	Yes	484 (3.4)	134 (2.9)	0.88 (0.70–1.10)						
Bench carpenters	No	13,770 (95.8)	4128 (96.4)							
	Yes	93 (0.6)	30 (0.7)	1.10 (0.69–1.74)						
Cabinetmakers and joiners	No	13,836 (96.3)	4144 (96.7)							
	Yes	27 (0.2)	14 (0.3)	2.06 (1.00–4.25)						
Woodworking machine operators	No	13,826 (96.2)	4153 (96.9)							
	Yes	37 (0.3)	5 (0.1)	0.54 (0.21–1.42)						
Woodworking occupations n.e.c.	No	13,814 (96.1)	4150 (98.9)							
	Yes	49 (0.3)	8 (0.2)	0.47 (0.20–1.11)						
*Mothers exposed to wood*										
Any wood job	No	6862 (66.8)	2065 (67.3)		1871 (54.9)	834 (64.4)		2821 (60.6)	1231 (69.5)	
	Yes	16 (0.2)	7 (0.2)	1.36 (0.48–3.86)	6 (0.2)	0 (0.00)	NA	9 (0.2)	7 (0.4)	1.93 (0.62–5.97)
Total		6878 (66.9)	2072 (67.5)		1877 (55.1)	834 (64.4)		2830 (60.8)	1238 (69.9)	

*CI* confidence interval; *N* number; *OR* odds ratio; *n.e.c.* not elsewhere classified

^a^Mothers and fathers not working were excluded

^b^Restricted to parents with occupational information the year − 1 and year of birth or the same job before and after birth

## Discussion

The present NORD-TEST analysis including Finland, Norway and Sweden showed no increased risk of TGCT in sons related to prenatal parental wood-related jobs or associated exposures. In contrast, sons of fathers working in “any wood-related job” and “woodworking occupations n.e.c.” had a decreased risk of TGCT, which was no longer statistically significant when the analyses were restricted to fathers with the same job in the census before and after birth, or census data available around childbirth (± 1 year). In this sensitivity analysis, sons of fathers working as “cabinetmakers and joiners” showed a marginally increased TGCT risk.

A previous analysis in NORD-TEST in Denmark showed an increased risk of TGCT in a small group of sons whose fathers were exposed to heavy metals other than lead (20). Most of these fathers were exposed to both chromium and toluene, and mainly working in wood-related jobs around the time of conception. We suspected that either the joint exposure to chromium and toluene or some other agents that are used in these jobs could explain the elevated TGCT risk in sons of this small group of fathers in Denmark. For example, pentachlorophenol, which is a phenolic organohalogen used for wood preservation, is suspected to interfere with human male genitalia development (Meijer et al. 2012). Even though such agents have been used in Finland, Norway and Sweden (Guvenius et al. 2003), combined exposure to chromium and toluene in wood-related jobs was not associated with an increased risk of TGCT in the current analysis. The reasons for the difference in results are unknown but may be due to random variation and the small numbers of exposed cases and controls.

Epidemiological studies have shown inconsistent results for both parental wood-related jobs as well as patients wood-related jobs (Knight and Marrett 1997; Pukkala et al. 2009; Stang et al. 2005). An American cohort showed an increased risk of testicular cancer in cabinetmakers and a decreased risk in carpenters (Dement et al. 2003). Our finding that “any wood-related job” and “woodworking occupations n.e.c.” associated with a decreased risk of TGCT are in line with a study in the US where sons of construction workers had a lower risk (Kardaun et al. 1991), but not with a study in Canada where sons of fathers who worked as wood processors had an increased risk of TGCT (Knight and Marrett 1997). The observed discrepancies seem to be partly due to the definition of jobs, which differ by job classification. The marginally increased risk we observed in “cabinetmakers and joiners” may be a chance finding in view of the large number of statistical tests performed. Yet, “cabinetmakers and joiners” can be exposed to lots of agents including formaldehyde, and wood preservatives containing chromium, copper, and arsenic, which show testicular toxicity in human and experimental studies (Duong et al. 2011; Rana 2014).

The present study has important strengths. It was conducted in the Nordic countries, a region known to have the highest incidence of testicular cancer in the world, with the Nordic population-based registries, known for their high quality of data registration (Pukkala et al. 2018), and their completeness (Hakulinen et al. 1986; Pukkala et al. 2018). The large sample size enabled an epidemiological study of a relatively rare cancer, but nevertheless, some exposures were rare, especially among mothers. Moreover, the use of a unique personal identification number in all Nordic countries allowed linkage of data between registries that provided accurate and valuable information on index persons as well as their relatives. This permitted us to describe the study population and assess potential confounding factors. Another strength was the use of the NOCCA-JEMs that allowed us to examine not only the association between job categories and TGCT in offspring but also specific occupational exposures.

Study limitations included potential exposure misclassification. Some misclassification of exposure is inevitable when using a JEM assuming no inter-individual variations of a certain exposure within the same occupation. Although the NOCCA-JEM provides the average proportion of people exposed (P) and the mean level of exposure (L) among exposed for each occupation, given that no actual measurement of exposure was performed for individual parents, exposure misclassification is likely, particularly when the prevalence (P) of exposure in a job is low. However, since the occupational information was collected prior to the son's diagnosis of TGCT, there is no reason to believe that degree or direction of exposure misclassification differed between cases and controls.

The use of census data is another source of exposure misclassification since the information relied on self-report, and misclassification of occupations may occur when censuses were not collecting data around the pregnancy period. The median time between the last census and birth was 3 years (interquartile range = 1–4). If there was any change of jobs in between the last census and childbirth, their exposures may be misclassified. To assess the potential bias introduced due to the missed information, we conducted a sensitivity analysis restricted to parents with occupational information during the year before or the year of birth, or those with the same information before and after birth. This sensitivity analysis revealed an increased risk of TGCT in sons of father’s working as “cabinetmakers and joiners”, while the remaining results were essentially identical to the findings from the primary analysis.

Another limitation was that the data from censuses did not allow differentiation between non-working parents (= non-exposed) and real missing data on occupation (= potentially exposed). Therefore, we were obliged to exclude all subjects with missing data for both parents (~ 10%) from the study, those with missing data for mothers (~ 50%) from the analysis of maternal exposure, and those with missing data for fathers (~ 14%) from the analysis of paternal exposure. A plausible reason for missing data is, e.g. graduate studies, especially among the young parents. The proportion of non-working parents was nevertheless similar among cases and controls, and therefore we do not think our results are biased due to this limitation.

Lastly, it should also be noted that the overall number of parents working in wood-related jobs was small (0.2% of the mothers and 4.8% of the fathers), resulting in imprecise estimates for some of the exposures in our study and limits the interpretation of association between parental occupation in wood-related jobs and TGCT in offspring, especially in mothers (*N* = 60). It also shows limited preventive potential for testicular cancer.

Improved exposure assessment, which is needed to support or refute our results, is challenging in registry-based case–control studies. Therefore, epidemiological studies on parental exposures may be complemented with experimental studies to further understand or refute potential biological effects of wood-related exposures, including wood preserving agents whereof formaldehyde.

## Conclusion

This large registry-based case–control study conducted in three Nordic countries provided no evidence of increased risk of TGCT in offspring associated with prenatal parental occupational exposures in wood-related jobs, with the exception of an increased risk in the small group of sons of ‘cabinetmakers and joiners’ in sensitivity analysis, which is supported by studies on testicular toxicity from chemicals used in this job.

## Supplementary Information

Below is the link to the electronic supplementary material.

Supplementary file1 (PDF 106 KB)

## Data Availability

The datasets generated during and/or analysed during the current study are not publicly available due to data privacy directives at the time of data being pooled, but are available from the corresponding author on reasonable request.

## References

[CR1] Archibong AE, Rideout ML, Harris KJ, Ramesh A (2018). Oxidative stress in reproductive toxicology. Curr Opin Toxicol.

[CR2] Baird DC, Meyers GJ, Hu JS (2018). Testicular cancer: diagnosis and treatment. Am Fam Physician.

[CR3] Béranger R, Le Cornet C, Schüz J, Fervers B (2013). Occupational and environmental exposures associated with testicular germ cell tumours: systematic review of prenatal and life-long exposures. PLoS ONE.

[CR4] Dement J, Pompeii L, Lipkus IM, Samsa GP (2003). Cancer incidence among union carpenters in New Jersey. J Occup Environ Med.

[CR5] Duong A, Steinmaus C, McHale CM, Vaughan CP, Zhang L (2011). Reproductive and developmental toxicity of formaldehyde: a systematic review. Mutat Res.

[CR6] Dutta S, Sengupta P, Slama P, Roychoudhury S (2021). Oxidative stress, testicular inflammatory pathways, and male reproduction. Int J Mol Sci.

[CR7] Ferlay J (2021). Cancer statistics for the year 2020: an overview. Int J Cancer.

[CR8] Feychting M, Plato N, Nise G, Ahlbom A (2001). Paternal occupational exposures and childhood cancer. Environ Health Perspect.

[CR9] Ghelli F (2021). Oxidative stress induction in woodworkers occupationally exposed to wood dust and formaldehyde. J Occup Med Toxicol.

[CR10] Gilligan T (2019). Testicular cancer, version 2. 2020, NCCN clinical practice guidelines in oncology. J Natl Compr Canc Netw.

[CR11] Guvenius DM, Aronsson A, Ekman-Ordeberg G, Bergman A, Norén K (2003). Human prenatal and postnatal exposure to polybrominated diphenyl ethers, polychlorinated biphenyls, polychlorobiphenylols, and pentachlorophenol. Environ Health Perspect.

[CR12] Hakulinen T, Andersen A, Malker B, Pukkala E, Schou G, Tulinius H (1986). Trends in cancer incidence in the Nordic countries. A collaborative study of the five Nordic Cancer Registries. Acta Pathol Microbiol Immunol Scand Suppl.

[CR13] Huyghe E, Plante P, Thonneau PF (2007). Testicular cancer variations in time and space in Europe. Eur Urol.

[CR14] Kardaun JW, Hayes RB, Pottern LM, Brown LM, Hoover RN (1991). Testicular cancer in young men and parental occupational exposure. Am J Ind Med.

[CR15] Kauppinen T (2009). Construction of job-exposure matrices for the Nordic Occupational Cancer Study (NOCCA). Acta Oncol.

[CR16] Knight JA, Marrett LD (1997). Parental occupational exposure and the risk of testicular cancer in Ontario. J Occup Environ Med.

[CR17] Lacson JC, Carroll JD, Tuazon E, Castelao EJ, Bernstein L, Cortessis VK (2012). Population-based case-control study of recreational drug use and testis cancer risk confirms an association between marijuana use and nonseminoma risk. Cancer.

[CR18] Le Cornet C (2017). Parental occupational exposure to organic solvents and testicular germ cell tumors in their offspring: NORD-TEST study. Environ Health Perspect.

[CR19] McGlynn KA, Trabert B (2012). Adolescent and adult risk factors for testicular cancer. Nat Rev Urol.

[CR20] Meijer L (2012). Influence of prenatal organohalogen levels on infant male sexual development: sex hormone levels, testes volume and penile length. Hum Reprod.

[CR21] Moch H, Cubilla AL, Humphrey PA, Reuter VE, Ulbright TM (2016). The 2016 WHO classification of tumours of the urinary system and male genital organs-part A: renal, penile, and testicular tumours. Eur Urol.

[CR22] Olsson A (2018). Parental occupational exposure to solvents and heavy metals and risk of developing testicular germ cell tumors in sons (NORD-TEST Denmark). Scand J Work Environ Health.

[CR23] Parekh NV, Lundy SD, Vij SC (2020). Fertility considerations in men with testicular cancer. Transl Androl Urol.

[CR24] Pereira SC, Oliveira PF, Oliveira SR, Pereira ML, Alves MG (2021). Impact of environmental and lifestyle use of chromium on male fertility: focus on antioxidant activity and oxidative stress. Antioxidants (basel).

[CR25] Pleskacova J (2010). Tumor risk in disorders of sex development. Sex Dev.

[CR26] Pukkala E (2009). Occupation and cancer—follow-up of 15 million people in five Nordic countries. Acta Oncol.

[CR27] Pukkala E (2018). Nordic cancer registries—an overview of their procedures and data comparability. Acta Oncol.

[CR28] Rana SV (2014). Perspectives in endocrine toxicity of heavy metals—a review. Biol Trace Elem Res.

[CR29] Schaffar R, Pant S, Bouchardy C, Schubert H, Rapiti E (2019). Testicular cancer in Geneva, Switzerland, 1970–2012: incidence trends, survival and risk of second cancer. BMC Urol.

[CR30] Schmiedel S, Schüz J, Skakkebaek NE, Johansen C (2010). Testicular germ cell cancer incidence in an immigration perspective, Denmark, 1978 to 2003. J Urol.

[CR31] Sharpe RM, Skakkebaek NE (1993). Are oestrogens involved in falling sperm counts and disorders of the male reproductive tract?. Lancet.

[CR32] Skakkebaek NE, Berthelsen JG, Giwercman A, Müller J (1987). Carcinoma-in-situ of the testis: possible origin from gonocytes and precursor of all types of germ cell tumours except spermatocytoma. Int J Androl.

[CR33] Skakkebaek NE, Rajpert-De Meyts E, Main KM (2001). Testicular dysgenesis syndrome: an increasingly common developmental disorder with environmental aspects. Hum Reprod.

[CR34] Sonne SB (2009). Analysis of gene expression profiles of microdissected cell populations indicates that testicular carcinoma in situ is an arrested gonocyte. Cancer Res.

[CR35] Stang A, Ahrens W, Baumgardt-Elms C, Bromen K, Stegmaier C, Jöckel KH (2005). Carpenters, cabinetmakers, and risk of testicular germ cell cancer. J Occup Environ Med.

[CR36] Sujatha R, Chitra KC, Latchoumycandane C, Mathur PP (2001). Effect of lindane on testicular antioxidant system and steroidogenic enzymes in adult rats. Asian J Androl.

[CR37] Togawa K (2016). Parental occupational exposure to heavy metals and welding fumes and risk of testicular germ cell tumors in offspring: a registry-based case-control study. Cancer Epidemiol Biomarkers Prev.

[CR38] Vallières E, Pintos J, Parent ME, Siemiatycki J (2015). Occupational exposure to wood dust and risk of lung cancer in two population-based case–control studies in Montreal, Canada. Environ Health.

[CR39] Znaor A, Lortet-Tieulent J, Jemal A, Bray F (2014). International variations and trends in testicular cancer incidence and mortality. Eur Urol.

