# Supplementary material for: Parental occupational exposures in wood-related jobs and risk of testicular germ cell tumours in offspring in NORD-TEST a registry-based case–control study in Finland, Norway, and Sweden
Source: Int Arch Occup Environ Health. 2021 Dec 1;95(6):1243–53. doi: 10.1007/s00420-021-01818-4 (PMC9273544; doi:10.1007/s00420-021-01818-4)
Supplement: Supplementary file 1 — Supplementary file1 (PDF 106 KB) [file 420_2021_1818_MOESM1_ESM.pdf]

Parental occupational exposures in wood-related jobs and risk of testicular germ cell tumours in offspring in NORD-TEST a registry-based case-control study in Finland, Norway, and Sweden

International Archives of Occupational and Environmental Health
https://doi.org./10.1007/s00420-021-01818-4

Sara Corbin, Kayo Togawa, Joachim Schüz, Charlotte Le Cornet, Beatrice Fervers, Maria Feychting, Pernilla Wiebert, Johnni Hansen, Susanne Oksbjerg Dalton, Kristina Kjærheim, Karl-Christian Nordby, Ragnhild Strand Østrem, Niels E. Skakkebæk, Sanni Uuksulainen, Eero Pukkala, Ann Olsson

Corresponding author: Dr. Ann Olsson, Environment and Lifestyle Epidemiology Branch, International Agency for Research on Cancer, World Health Organization, 150 cours Albert Thomas, 69372 Lyon CEDEX 08, France. E-mail: olssona@iarc.fr

Supplementary Table 1. Specific exposures assigned to wood-related jobs in the NOCCA-JEM

Wood-related jobs	***Exposures***	***Other occupations exposed***
Upholsterers<br>Timbermen<br>Sawyers<br>Plywood and fibreboard workers<br>Construction carpenters<br>Wooden boatbuilders, coach-body builders etc.<br>Bench carpenters<br>Cabinetmakers and joiners etc.<br>Woodworking machine operators etc.<br>Wooden surface finishers<br>Woodworking occupations, n.e.c.	Wood dust	
Upholsterers	Benzene	Chemists<br>Laboratory assistants<br>Service station attendants<br>Well drilling and quarrying<br>Leather cutters for footwear<br>Shoe sewers<br>Lasters and sole fitters etc.<br>Footwear workers, nec<br>Machine and engine mechanics<br>Painters, lacquerers and floor layers
Upholsterers<br>Woodworking occupations, n.e.c.	Toluene	Service station attendants<br>Leather cutters for footwear<br>Shoe sewers<br>Lasters and sole fitters etc.<br>Footwear workers, nec<br>Machine and engine mechanics<br>Painters, lacquerers and floor layers

Upholsterers	Methylene chloride	Lasters and sole fitters etc.<br>Machine and engine mechanics<br>Electronics and telecommunications workmen<br>Electrical and electronic equipment assemblers<br>Painters, lacquerers and floor layers
Upholsterers	1,1,1-Trichloroethane	Lasters and sole fitters etc.<br>Occupations in smelting, metallurgical and foundry work, nec<br>Turners, toolmakers and machine-tool setters<br>Machine and engine mechanics<br>Metal plating and coating work<br>Assemblers and other machine and metalware occupations<br>Electronics and telecommunications workmen<br>Electrical and electronic equipment assemblers<br>Painters, lacquerers and floor layers
Upholsterers<br>Wooden surface finishers	Aliphatic and alicyclic hydrocarbon solvents	Leather cutters for footwear<br>Shoe sewers<br>Lasters and sole fitters etc.<br>Footwear workers, nec<br>Machine and engine mechanics<br>Painters, lacquerers and floor layers
Upholsterers<br>Bench carpenters	Aromatic hydrocarbon solvents	Chemists<br>Laboratory assistants<br>Service station attendants<br>Leather cutters for footwear<br>Shoe sewers<br>Lasters and sole fitters etc.<br>Footwear workers, nec<br>Painters, lacquerers and floor layers
Sawyers<br>Plywood and fibreboard workers<br>Woodworking machine operators etc.<br>Wooden surface finishers<br>Woodworking occupations, n.e.c.	Chromium	Metal smelting furnacemen<br>Heat treaters, hardeners, temperers etc.<br>Cold- and hot-rolling metal workers<br>Smiths<br>Foundry workers<br>Wire and pipe drawers<br>Occupations in smelting, metallurgical and foundry work, nec<br>Turners, toolmakers and machine-tool setters<br>Fitter-assemblers etc.<br>Machine and engine mechanics<br>Sheet metal workers<br>Plumbers<br>Welders and flame cutters<br>Metal plating and coating work<br>Assemblers and other machine and metalware occupations<br>Painters, lacquerers and floor layers

Sawyers<br>Plywood and fibreboard workers	Fungicides	Farmers, silviculturists, horticulturists<br>Commercial garden and park workers<br>Forestry workers and lumberjacks<br>Cookers and furnacemen (chemical processes)
Plywood and fibreboard workers<br>Wooden boatbuilders, coach-body builders etc.<br>Bench carpenters<br>Cabinetmakers and joiners etc.<br>Woodworking machine operators etc.<br>Wooden surface finishers<br>Woodworking occupations, n.e.c.	Formaldehyde	Technical nursing assistants<br>Livestock breeders<br>Forestry workers and lumberjacks<br>Textile finishers, dyers<br>Textile inspectors<br>Patternmakers and cutters (also leather garments and gloves)<br>Industrial sewers etc. (also leather garments and gloves)<br>Foundry workers<br>Welders and flame cutters<br>Metal plating and coating work<br>Electronics and telecommunications workmen<br>Painters, lacquerers and floor layers
Plywood and fibreboard workers	Insecticides	Farmers, silviculturists, horticulturists<br>Commercial garden and park workers
Construction carpenters	Quartz dust	Miners, shot firers etc.<br>Well drilling and quarrying<br>Concentration plant workers<br>Miners and quarrymen, nec<br>Metal smelting furnacemen<br>Foundry workers<br>Occupations in smelting, metallurgical and foundry work, nec<br>Bricklayers, plasterers and tile setters<br>Reinforced concretelayers, stonemasons etc.<br>Concrete shutterers and finishers
Construction carpenters	Asbestos	Miners, shot firers etc.<br>Concentration plant workers<br>Miners and quarrymen, nec<br>Railway engine drivers, steam engine firemen<br>Metal smelting furnacemen<br>Fitter-assemblers etc.<br>Machine and engine mechanics<br>Sheet metal workers<br>Plumbers<br>Welders and flame cutters<br>Electricians<br>Electronics and telecommunications workmen<br>Painters, lacquerers and floor layers<br>Bricklayers, plasterers and tile setters
